# Left atrium 4D-flow segmentation with high-resolution contrast-enhanced magnetic resonance angiography

**DOI:** 10.3389/fcvm.2023.1225922

**Published:** 2023-10-09

**Authors:** Hansuk Kim, Stephen B. Wilton, Julio Garcia

**Affiliations:** ^1^ Biomedical Engineering, University of Calgary, Calgary, AB, Canada; ^2^ Stephenson Cardiac Imaging Centre, University of Calgary, Calgary, AB, Canada; ^3^ Libin Cardiovascular Institute, University of Calgary, Calgary, AB, Canada; ^4^ Department of Cardiac Sciences, University of Calgary, Calgary, AB, Canada; ^5^ Department of Radiology, University of Calgary, Calgary, AB, Canada; ^6^ Alberta Children’s Hospital Research Institute, University of Calgary, Calgary, AB, Canada

**Keywords:** 4D-flow, segmentation, left atrium, CE-MRA, registration, stasis

## Abstract

**Background:**

Atrial fibrillation (AF) leads to intracardiac thrombus and an associated risk of stroke. Phase-contrast cardiovascular magnetic resonance (CMR) with flow-encoding in all three spatial directions (4D-flow) provides a time-resolved 3D volume image with 3D blood velocity, which brings individual hemodynamic information affecting thrombus formation. As the resolution and contrast of 4D-flow are limited, we proposed a semi-automated 4D-flow segmentation method for the left atrium (LA) using a standard-of-care contrast-enhanced magnetic resonance angiography (CE-MRA) and registration technique.

**Methods:**

LA of 54 patients with AF were segmented from 4D-flow taken in sinus rhythm using two segmentation methods. (1) Phase-contrast magnetic resonance angiography (PC-MRA) was calculated from 4D-flow, and LA was segmented slice-by-slice manually. (2) LA and other structures were segmented from CE-MRA and transformed into 4D-flow coordinates by registration with the mutual information method. Overlap of volume was tested by the Dice similarity coefficient (DSC) and the average symmetric surface distance (ASSD). Mean velocity and stasis were calculated to compare the functional property of LA from two segmentation methods.

**Results:**

LA volumes from segmentation on CE-MRA were strongly correlated with PC-MRA volume, although mean CE-MRA volumes were about 10% larger. The proposed registration scheme resulted in visually successful registration in 76% of cases after two rounds of registration. The mean of DSC of the registered cases was 0.770 ± 0.045, and the mean of ASSD was 2.704 mm ± 0.668 mm. Mean velocity had no significant difference between the two segmentation methods, and mean stasis had a 3.3% difference.

**Conclusion:**

The proposed CE-MRA segmentation and registration method can generate segmentation for 4D-flow images. This method will facilitate 4D-flow analysis for AF patients by making segmentation easier and overcoming the limit of resolution.

## 1. Introduction

Atrial fibrillation (AF) is a growing epidemic affecting over 37 million individuals worldwide in 2017, and the number of individuals with AF will increase at least 60% by 2050 (1). AF is a heart condition that causes an irregular and often very rapid heart rhythm, leading to blood clots in the heart and increasing the risk of stroke, heart failure and mortality (2, 3). Currently, the CHA_2_DS_2_-VASc risk score based on clinical risk factors is widely used for assessing the risk of stroke and choosing appropriate antithrombic therapy in patients with AF (4–6). However, its prediction accuracy is limited as it does not count individual hemodynamic patterns affecting thrombus formation. Cross-sectional transesophageal echocardiography studies have indicated the association of flow velocity in the left atrium (LA) and left atrial appendage (LAA) with thromboembolic risk in patients with AF (7, 8). Furthermore, phase-contrast cardiovascular magnetic resonance (CMR) with flow-encoding in all three spatial directions (4D-flow) provides 3D blood velocity information in 3D volume throughout the cardiac cycle (9–11). With its power of visualization and quantification, this technique opened new horizons in understanding cardiovascular flow and has been used to assess flow patterns and parameters, including velocity, stasis, and vorticity in LA and LAA in patients with AF (12–22). However, the segmentation of heart chambers is challenging because of the limited resolution and contrast of the 4D-flow. Many 4D-flow scan parameters have a trade-off with scan time. The conventional scan takes about 10 min for a 3 mm resolution, which provides insufficient detail for the segmentation of LAA in many cases. In addition, during 4D-flow acquisition after routine measurements, wash-out of the contrast agent further reduces the contrast of 4D-flow. Thus, most previous 4D-flow studies in AF patients relied upon manual segmentation on magnitude image (13, 20) or phase-contrast magnetic resonance angiography (PC-MRA) (12, 13, 15, 16, 18, 21, 22), which is time-consuming, depends on the operator's knowledge and experience, and has limited reproducibility (20). An interesting approach was 4D-flow co-registration with the cine images (19). However, it requires a non-standard extra cine sequence covering the complete volume of the LA.

Contrast-enhanced magnetic resonance angiography (CE-MRA) is part of the standard-of-care imaging protocol for patients referred for cardiovascular disease. With an injection of gadolinium contrast agent, which shortens the T1 relaxation time, the image of the vascular system is acquired in 3D with enhanced resolution (−1 mm). Moreover, CE-MRA can benefit from the optimal timing of contrast agent injection. Therefore, fine structure in the heart can be readily recognized with CE-MRA without the need for additional CMR sequences.

This study proposes a semi-automated segmentation method for LA by using enhanced contrast and resolution of the CE-MRA image. We hypothesized that the registration of CE-MRA and PC-MRA would transform segmentations from CE-MRA to 4D-flow coordinates, and this transformed segmentation can yield compatible analysis results from the conventional PC-MRA segmentation method.

## 2. Methods

### 2.1. Subject population

**Figure 1:**
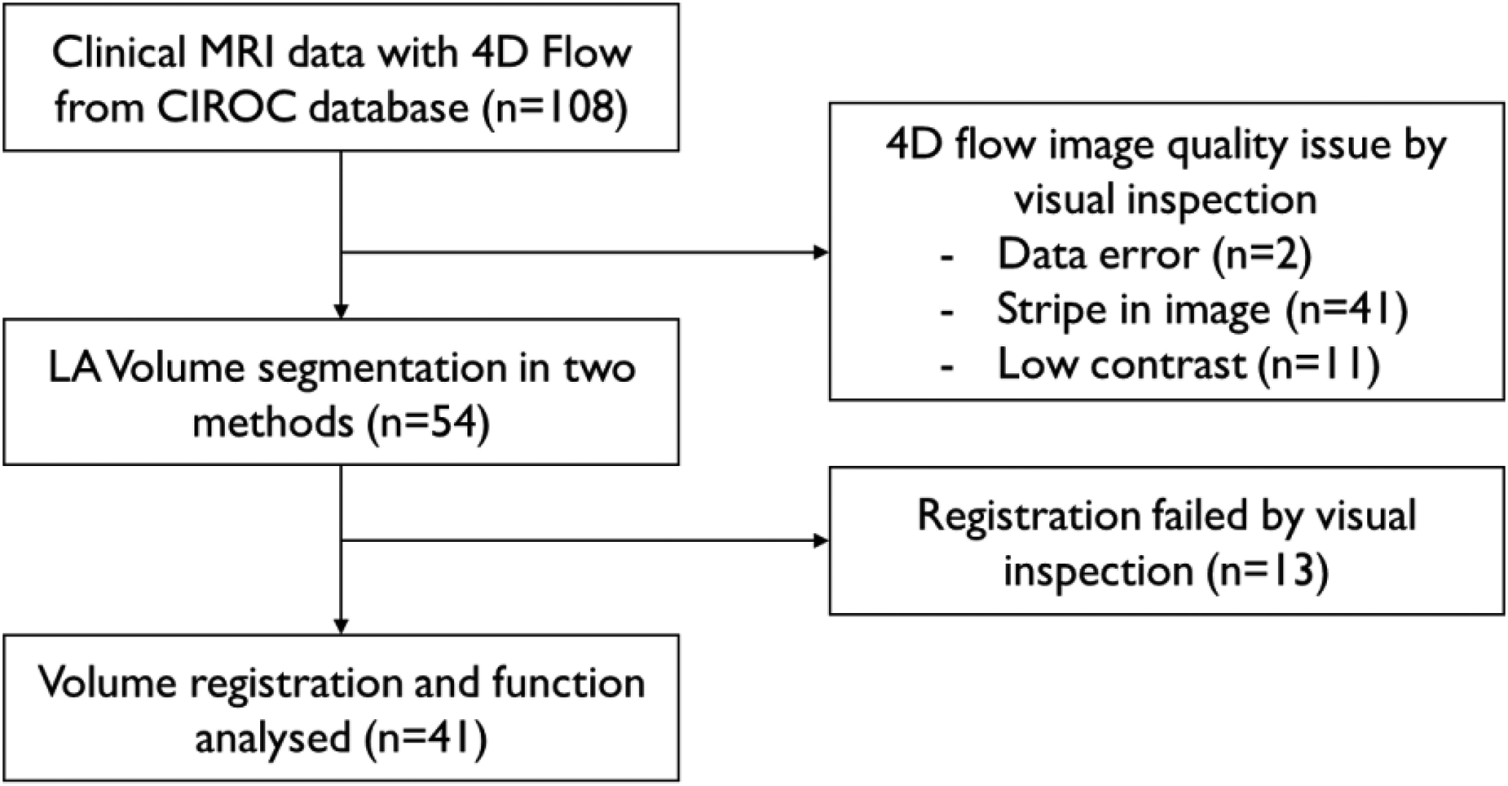
Decision tree for subject inclusion and exclusion. Patients with 4D-flow MRI scan from CIROC database were included. After excluding apparent quality issue data, volume segmentation of the two methods was assessed. Cases were evaluated after registration. Apparently failed cases were excluded from the final analysis.

**Figure 2:**
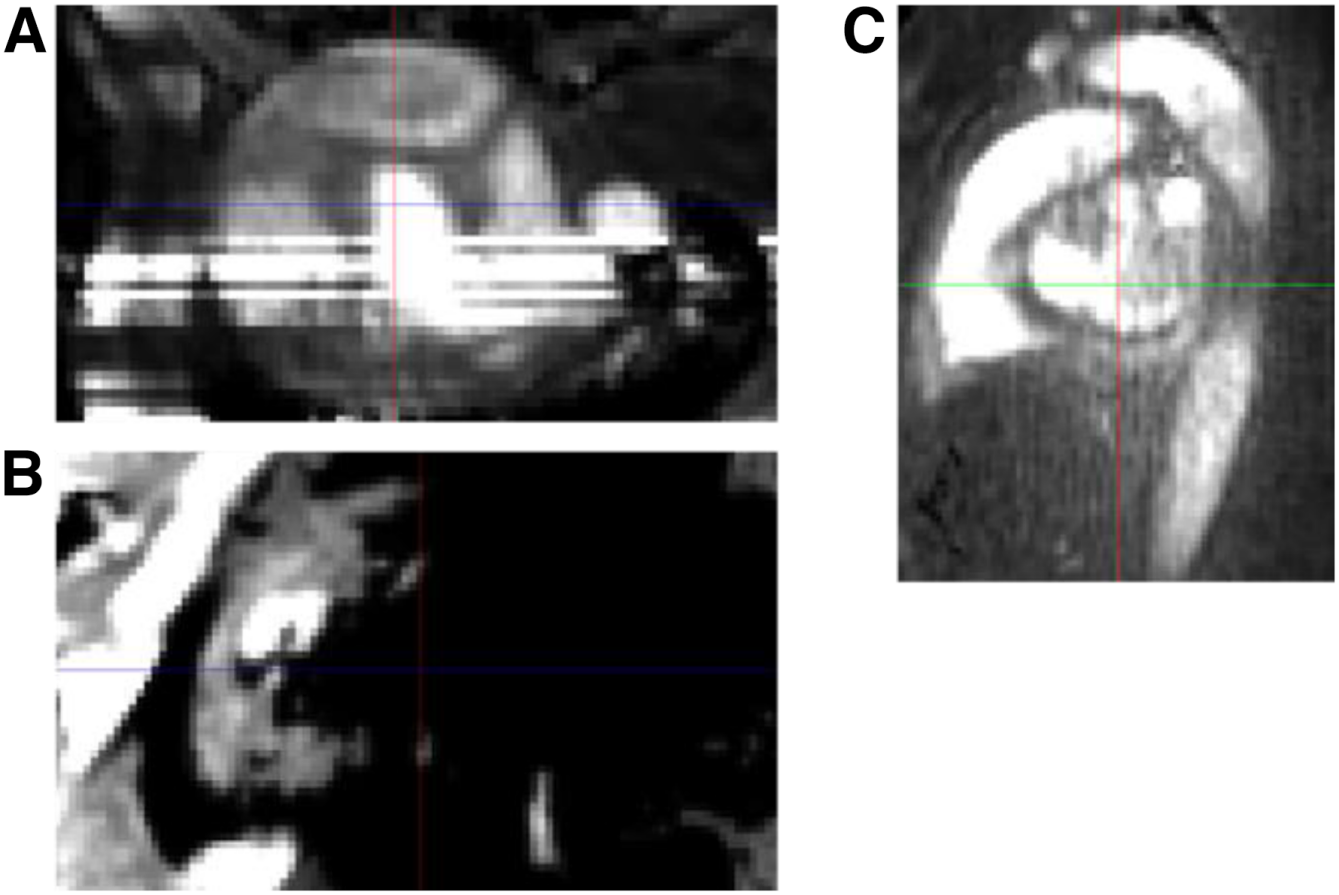
PC-MRA image example of excluded cases due to 4D-flow image quality issue. (**A**) Stripe. (**B**) Partly void due to low intensity. (**C**) Low contrast noisy image.

In this retrospective study, a total of 108 patients with paroxysmal AF with normal systolic function, scanned between June 2017 and January 2020 with a 4D-flow scan, were retrospectively included from the Cardiovascular Imaging Registry of Calgary (CIROC) database (Figure 1). There were 54 cases excluded due to poor data quality in MR acquisition (Figure 2). In detail, there were data errors in 2 cases and stripes in magnitude and velocity images in 41 cases, and 11 cases were excluded due to low contrast. Fifty-four cases were accepted for LA volume segmentation (age = 56.4 ± 11.1, female = 16).

A commercial software (intakeDI™, Cohesic Inc., Calgary, Canada) was used to coordinate routine capture of patient informed consent and health questionnaires and for standardized collection of MRI-related variables. Patients with significant mitral or aortic valve disease and inappropriate/incomplete image reconstruction cases were not included. The study was approved by the University of Calgary's Conjoint Health Research Ethics Board, and all subjects gave written informed consent. All performed research activities were in accordance with the Declaration of Helsinki.

### 2.2. MRI data acquisition

All subjects were required to be in sinus rhythm at the time of CMR imaging. Patients and healthy subjects underwent an identical standardized MRI protocol using 3 T MR scanners Skyra/Prisma (Siemens, Erlangen, Germany) inclusive of standard multi-planar steady-state free-precession (SSFP) cine imaging in 4-chamber, 3-chamber, 2-chamber, short-axis of the LV at end-expiration. A three-dimensional magnetic resonance angiography (MRA) of LA was performed using the administration of 0.2 mmol/kg gadolinium contrast (Gadovist®, Bayer Inc., Mississauga, Ontario, Canada) at 2–3 ml/s. Three volumetric acquisitions were performed: one pre-contrast (for subtraction), one during the first pass, one after contrast administration. Subtraction volume was used for LA segmentation. Acquisition parameters were: Acquisition matrix = 384 × 286; repetition time = 3.39 ms; echo time = 1.16; flip angle = 20–25 degrees; and spatial resolution = 1.17 × 1.17 × 1.19–1.39 mm.

Approximately 5–10 min following contrast injection, 4D-flow MRI using an ECG retrospectively triggered sequence with respiratory navigator-based gating (WIP 785A) was taken with imaging parameters previously described (14, 18, 22): gating = prospective, flip angle = 15 degrees, FOV = 200–420 mm × 248–368 mm, spatial resolution = 2.0–3.5 × 2.0–3.5 × 2.5–3.5 mm; temporal resolution = 25–35 ms, 25–30 phases, and velocity sensitivity = 150–200 cm/s. Total acquisition time varies between 8 and 12 min, depending on heart rate and respiratory navigator efficiency.

### 2.3. Standard cardiac analysis and 4D-flow MRI pre-processing

A commercial software (cvi42 v5.11, Circle Cardiovascular Imaging Inc., Calgary, Canada) was used to determine the heart function from standard ECG-gated cine images. The short axis cine images were used to obtain LV end-diastolic volume (EDV), LV end-systolic volume (ESV), LV stroke volume (SV), LV mass, LV cardiac output (CO), LVEDV indexed to BSA, LVESV indexed to BSA, LV mass indexed to BSA and LV ejection fraction (LVEF).

4D-flow MRI pre-processing was performed using an in-house program developed in Matlab. Preprocessing steps included noise masking, velocity anti-aliasing, and corrections for Maxwell terms and eddy currents (22–24).

### 2.4. Conventional segmentation method

All data underwent preprocessing to correct for Maxwell terms, eddy current-induced phase offset, and velocity aliasing, if necessary. PC-MRA was generated from 4D-flow MRI by multiplying the magnitude image and the magnitude of velocity from the phase image for each voxel (23).

$$\mathrm{PCMRA}(\vec{r})=\;\frac{1}{N}\overset{N}{\underset{i=1}{\sum }}I_{i}^{Mag}(\vec{r})\sqrt{v_{x,i}^{2}(\vec{r})+\;v_{y,i}^{2}(\vec{r})+\;v_{z,i}^{2}(\vec{r})}$$

Where $\vec{r}$ is the spatial location within the volume, *i* is the measured time frame within the cardiac cycle out of a total number of *N* frames. Magntide and velocity images are noted by $I_{i}^{Mag}$ and *v*, velocity encoding direction is given by *x*, *y*, *z*. LA was segmented slice-by-slice manually using in-house software written in Matlab [9.12.0.1956245 (R2022a) The MathWorks Inc., Natick, Massachusetts, USA].

### 2.5. CE-MRA registration method

**Figure 3:**
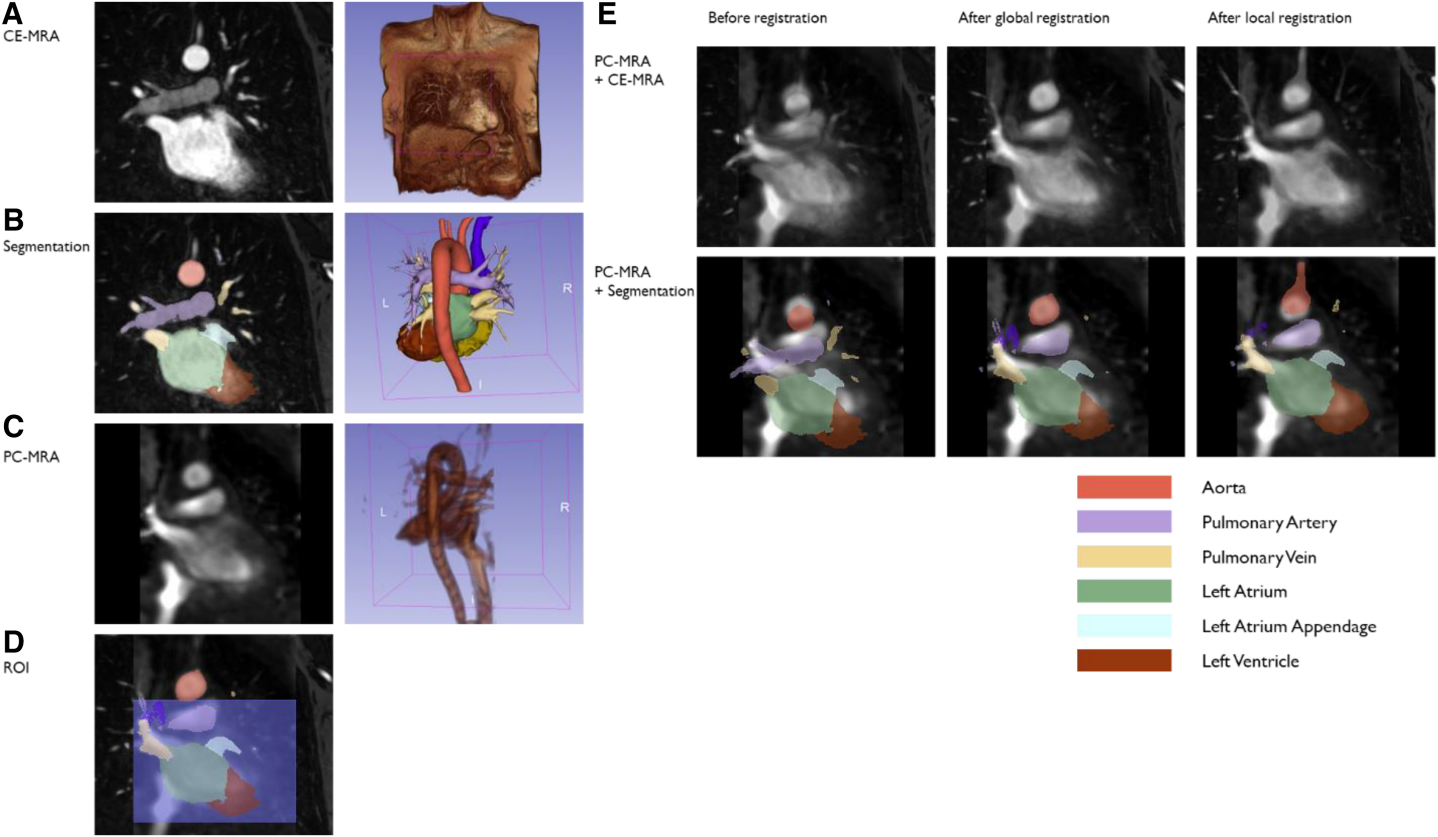
A sample case of the CE-MRA registration method. Segmentation was done on the high-resolution CE-MRA image (**A,B**). PC-MRA (**C**) was overlayed on CE-MRA, and registration was processed (**E**, top). CE-MRA segmentation was transformed following the same transform (**E**, bottom). The first round of registration may match the aortic arch rather than the heart chambers due to the difference in distance between them in the two images (**E**, middle column). For these cases, ROI was set around the heart chamber (**D**), and registration was processed once more (**E**, right column).

Figure 3 summarize the CE-MRA registration process. CE-MRA DICOM images and PC-MRA were imported into 3D Slicer 5.0.2 (25). Major vascular and chambers were segmented from CE-MRA images using the seed-and-grow method (Figure 3B). Seed points were given to LA as well as other major structures such as pulmonary vein (PV), pulmonary artery, aorta, and left ventricle for reference. Segments were refined interactively after initial growth. LAA was separated from LA by cutting at the orifice. A median filter and closing volume filter with a kernel size of 3 mm were applied for each segment. PC-MRA image generated from 4D flow from the previous section was also imported and underwent bias correction filter (Figure 3C).

General Registration (BRAINS) module (26) was used for finding the transformation of registering CE-MRA (moved) into PC-MRA image (fixed) (Figure 3E Top). As a cost metric, mutual information was chosen for its immunity in multi-modal registration. Rigid transformation was applied assuming there is no deformation between two measurements other than patient moving. Percentage of samples was set to 0.2 considering trade-off with registration quality and time consumption. Maximum iteration was set to 1,500 but not reached in any case. The same transformation was applied to CE-MRA segmentation to be overlaid on PC-MRA (Figure 3E Bottom). If the transformed LA and PV segments did not match well with the PC-MRA image by visual inspection, the second round of registration was executed with the region-of-interest (ROI), including the upper chambers but excluding the aortic arch (Figure 3D), and the percentage of samples was 1.0. ROI is set as a simple cube shape to keep the process simple and quick.

Finally, inspection-passed segmentations were transformed and resampled to be conformed with 4D-flow coordinates using in-house software written in Matlab. Nearest-neighbor interpolation method is used in resampling to generate binary mask.

### 2.6. Evaluation

The volume of the LA was measured from segmentations from PC-MRA and CE-MRA, respectively. The LA volume from CE-MRA is measured after transformation and resampling to PC-MRA coordinate space in accordance with the resolution of PC-MRA.

Registration of CE-MRA segmentation to 4D-flow was assessed by Dice similarity coefficient (DSC) (27) and average symmetric surface distance (ASSD) (28) with PC-MRA segmentation. DSC measures the spatial overlap between two regions.

$$\mathrm{DSC}\;(A,B)=\frac{2\;|A\cap B|}{\left|A|+|B\right|}$$

ASSD is the average distance from all surface voxels from both volumes.

$$\mathrm{ASSD}\;(A,B)=\frac{1}{\left|S(A)|+|S(B)\right|}\left(\underset{a\in S(A)}{\sum }\underset{b\in S(B)}{\min}\|a-b\|+\underset{b\in S(B)}{\sum }\underset{a\in S(A)}{\min}\|a-b\|\;\right)$$

where S(A) is the set of surface voxels of A and || || denotes Euclidean distance.

Mean velocity and stasis were calculated to compare the functional properties of LA from the two segmentation methods. For stasis, the velocity threshold was set to 0.1 m/s based on previous studies (15).

### 2.7. Statistics

Statistics were analyzed using IBM SPSS Statistics for Windows, version 29.0 (IBM Corp., Armonk, N.Y., USA). Shapiro-Wilk test of normality was conducted then variables were reported as mean ± standard deviation (SD) if normally distributed or median [interquartile range]. For volume comparisons, a two-tailed paired t-test was used to evaluate differences. Pearson's correlation method was used to determine the correlation between two variables. Bland–Altman analysis was reported with the mean differences and 95% confidence intervals for the limits of agreement.

## 3. Results

### 3.1. Subject demographics

Demographics of LA volume segmentation subjects are summarized in Table 1. The mean age was 56.4 years, and 30% were female. The median of the CHA_2_DS_2_-VASc score was 1.0. Detailed risk factors are described in Table 2. Among the subjects whose medical history records were available, hypertension, female, and age were common risk factors, and the overall risk score was low (=0) or moderate (=1) in 73%.

**Table 1:** Baseline characteristics.

	LA segmented (*n* = 54)
	Mean ± SD, Median [range] or count (percent)
Age (years)	56.4 ± 11.1
Sex (female)	16 (30%)
Height (m)	1.76 ± 0.10
Weight (kg)	84.5 [75.0–106.7]
BSA (m^2^)	2.09 ± 0.27
HR (bpm)	64 ± 12
Systolic BP (mmHg)	117 ± 15
Diastolic BP (mmHg)	69 ± 10
CHA_2_DS_2_-VASc	1.0 [0.0–2.0]

**Table 2:** Risk score and factors.

	LA segmented (valid *n* = 52)	
CHA_2_DS_2_-VASc		
0	16	31%
1	22	42%
2	10	19%
3	4	8%
Risk factors		
CHF/LV dysfunction	1	2%
Hypertension	18	35%
Aged 75 or over	0	0%
Diabetes	3	6%
Stroke	3	6%
Vascular disease	1	2%
Aged 65–74	13	25%
Sex category female	15	29%

CHF, congestive heart failure; LV, left ventricle.

### 3.2. Volume comparison

**Figure 4:**
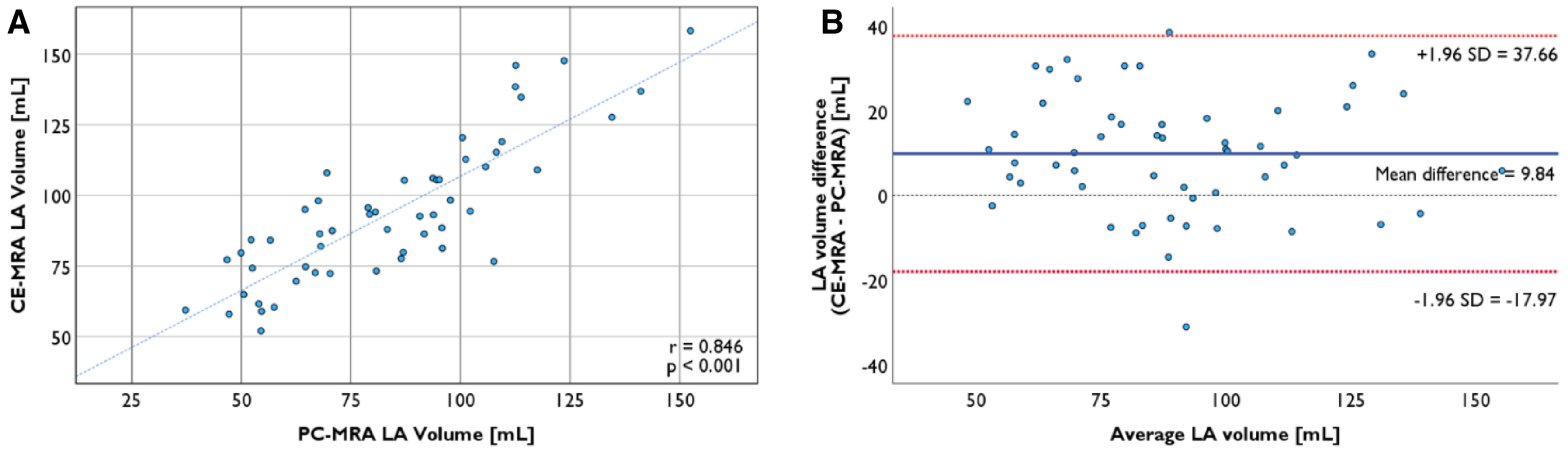
(**A**) LA segmentation from CE-MRA is strongly correlated with the segmentation from PC-MRA (*r* = 0.846, *p* < 0.001). (**B**) Bland-Altman plot of LA volume from two segmentation methods with the 95% limits of agreement.

LA volume measured from CE-MRA segmentation was 93.9 ± 24.9 ml, while volume from PC-MRA was 84.1 ± 26.1 ml (*p* < 0.001). The volume ratio of the two segmentations was 1.24 ± 0.26, and the two volumes have a strong positive association (*r* = 0.846, *p* < 0.001, Figure 4A). Bland-Altman plots revealed no proportional bias between the two segmentation methods' volume, and most of the data points lay in 95% limits of agreement (LoA) (Figure 4B). LAA volume from CE-MRA segmentation was 3.99 [2.68–6.13] ml.

### 3.3. Registration and overlap

**Figure 5:**
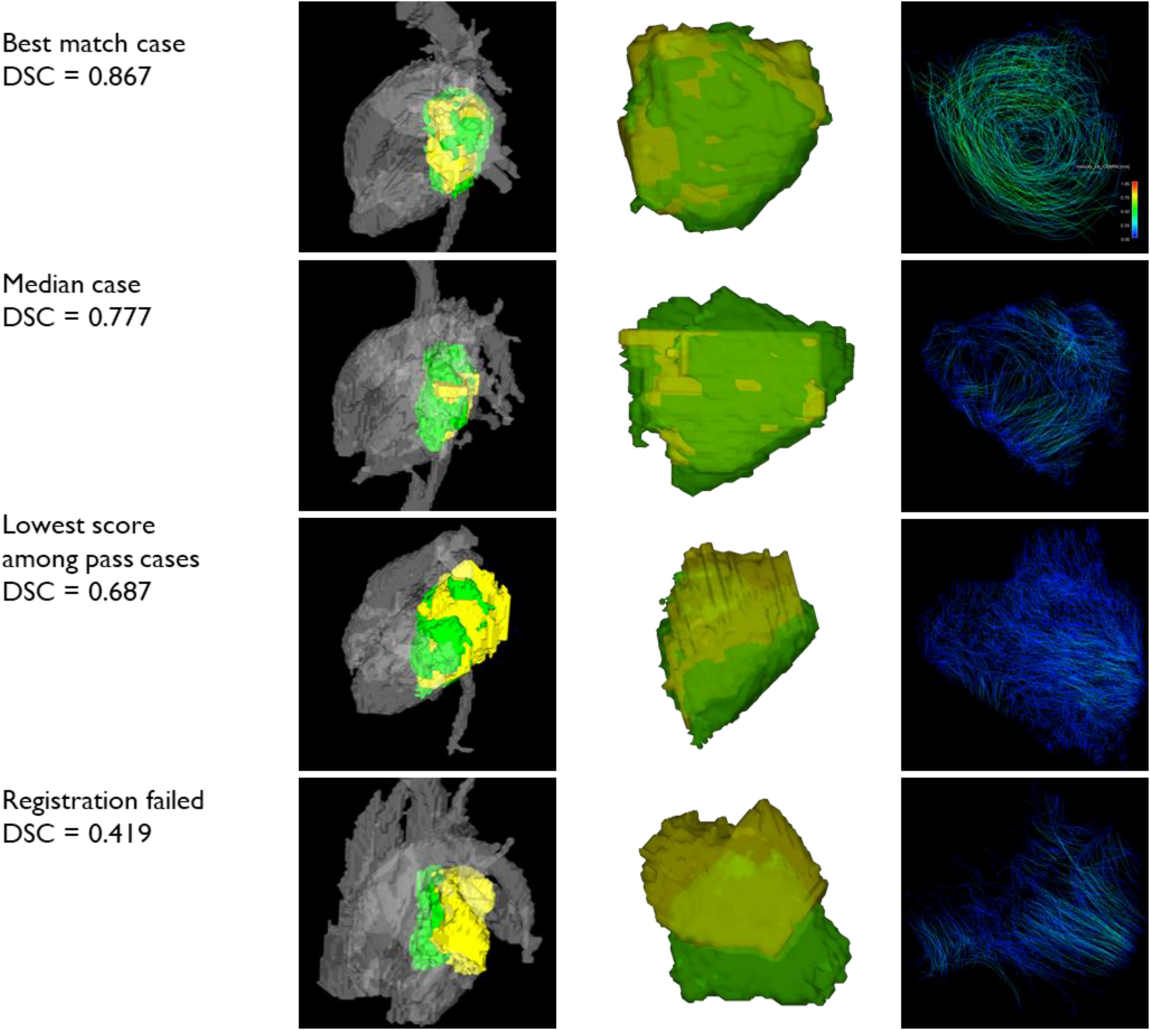
Samples of registration. Three successful registration cases with different Dice similarity scores and one rejected case are shown here. The left column shows LA segmentations with adjacent flows. The middle column shows two segmentations only. The right column shows the pathlines of LA flow. yellow: PC-MRA LA segmentation; green: CE-MRA LA segmentation.

The sample cases of registration are shown in Figure 5. The first round of registration of CE-MRA onto PC-MRA was successful in 22 cases by visual inspection out of 54 cases. The second round of registration was executed with ROI around LA for the other cases. Visual inspection accepted 19 cases while rejecting the other 13 cases. In all, 41 out of 54 (76%) cases of CE-MRA segmentation were successfully registered to the 4D-flow domain with this two-round scheme.

**Figure 6:**
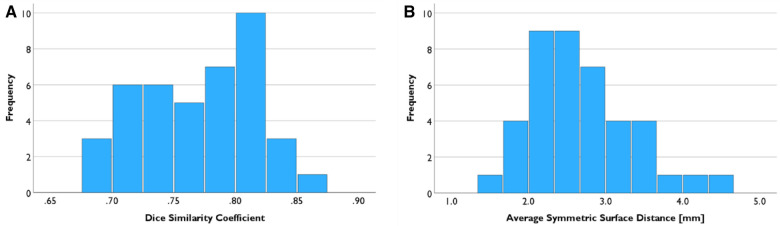
Registration result evaluation. (**A**) Mean of the Dice similarity coefficient between LA segmentation from PC-MRA and CE-MRA is 0.770. (**B**) Mean of the Average Symmetric Surface Distance was 2.704 mm.

The mean of DSC of the registered cases was 0.770 ± 0.045, and the mean of ASSD was 2.704 mm ± 0.668 mm (Figure 6). Shapiro–Wilk test results indicated that both data are normally distributed (*p* = 0.426, 0.093, respectively).

### 3.4. Functional evaluation in LA

**Figure 7:**
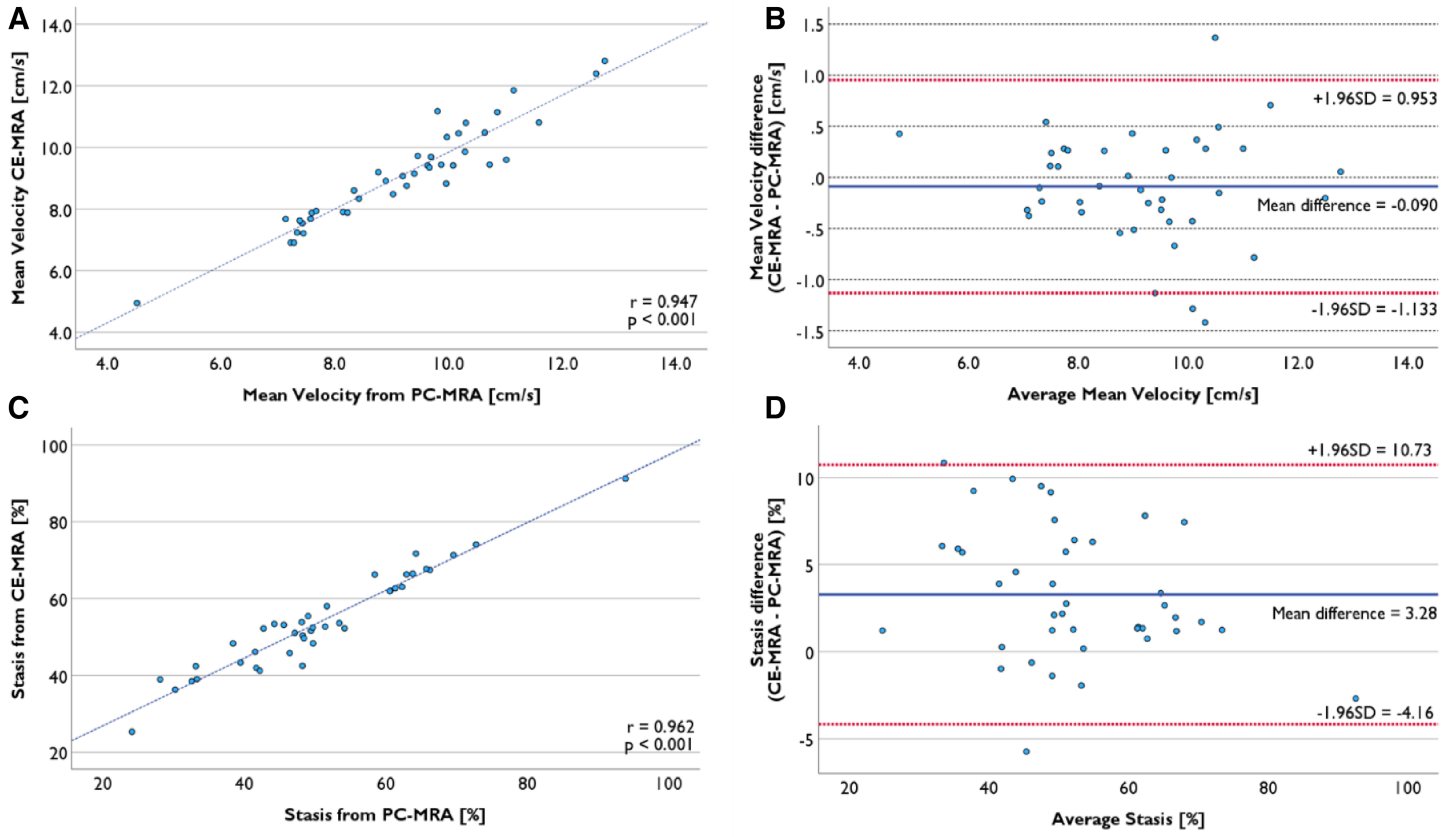
Comparison of functional evaluation using LA volume segmented from PC-MRA and CE-MRA. (**A**) Mean velocity from two volumes was strongly correlated. (**B**) Bland-Altman plot of mean velocity presents no proportional bias. (**C**) Mean stasis from two volumes was strongly correlated. (**D**) Mean difference was 3.28%, and there was no proportional bias.

There was no significant difference in the average mean velocity in LA volume from CE-MRA with mean velocity from PC-MRA segmentation (9.1 ± 1.6 cm/s vs. 9.2 ± 1.7 cm/s, *p* = 0.286) in 41 successful registration cases. Individual data points were strongly correlated (*r* = 0.947, *p* < 0.001, Figure 7A). No proportional bias was observed in the Bland–Altman plot (Figure 7B).

The average mean stasis calculated from LA volume from CE-MRA segmentation was 53.9 ± 12.6%, while the average mean stasis from PC-MRA was 50.6 ± 13.7%. There was a significant mean difference of 3.3% (*p* < 0.001). Nevertheless, the two datasets were strongly correlated (*r* = 0.962, *p* < 0.001), and there was no proportional bias (Figures 7C,D).

## 4. Discussion

In this study, we have developed a segmentation and registration workflow to use CE-MRA imaging data for 4D-flow analysis. Prior to analysis, a data quality check validated 50% of the total cases, while others were rejected due to limited 4D-flow data quality. LA volumes from segmentation on CE-MRA correlated strongly with PC-MRA volumes, although they were approximately 10% larger. The proposed registration scheme resulted in visually successful registration in 76% of cases. Despite volume differences and registration errors, mean velocity had no significant difference, while stasis had a significant but only 3.3% difference.

The measured LA volume was 93.9 ± 24.9 ml from CE-MRA segmentation and 84.1 ± 26.1 ml from PC-MRA segmentation. This value was reasonable for this AF cohort compared to AF-sinus cases in previous studies of 115 ± 42 ml and 64 ± 29 ml, repectively (15, 16). Volume was relatively largely evaluated in CE-MRA. This may come from different segmentation methods; the seed-and-grow method may include more partially-occupied voxels at the blood pool and tissue border than manual contouring. We counted the number of voxels on the surface of the LA segment. The average ratio of surface voxels to total LA voxels was 14% with 6 connectivity or 23% with 26 connectivity. This supports the decision of a boundary voxel can produce the difference between the two methods.

Registration of CE-MRA and PC-MRA was successful in 41% of cases on the first attempt. Among the registration parameters, the percentage of samples was adjusted to optimize registration quality and processing time. Varying other parameters did not affect the result. In many cases, the registration of the entire area corresponded to the aorta rather than the heart chambers. PC-MRA was generated by averaging over the whole cardiac cycle, whereas CE-MRA was taken from the target phase so that it may result in a relative positional difference between the aorta and the heart chambers. By excluding the aortic arch from the registration calculation, the LA was successfully registered in 19 out of 32 cases on the second registration attempt. Precise contouring of the ROI could further improve the success rate, but was not attempted because the purpose of this procedure is to keep it simple and easy. The average ASSD of 2.70 mm, which is less than one voxel length in 4D-flow, indicated that the registration result was acceptable. This study registered 76% of cases successfully after two rounds of registration. A previous study indicated success rate of mutual information was reduced with increase of noise level and this can be improved with modified cost function (29). Enhanced registration method including this approach would be applied in the following study.

In the functional evaluation, there was a 3.3% increase in mean stasis with CE-MRA while there was no significant difference in mean velocity. This increase in stasis may be related to the oversegmented volume in CE-MRA resulting from the boundary between the flow and stationary tissue. Due to the partial volume effect, these voxels should represent the velocity between flow velocity and tissue motion. With mean velocity, this effect is diluted in proportion to the total volume. Stasis, however, compares the velocity to a predefined threshold and converts it to binary, thus exaggerating partial volume voxels. Nevertheless, this difference in stasis has no practical impact on 4D-flow analysis, as the stasis difference between AF patients and healthy controls was 10% to 18% in previous studies (15, 16, 18, 19).

Approximately, PC-MRA manual segmentation took 20–30 min per case. All cases were performed by a 4-year 4D-flow MRI reader specialized in AF. In comparison, CE-MRA seed-and-grow segmentation took around 10–15 min, and automatic registration computation time was about 15–20 s for the first round and 35–50 s for the second round using an Intel Core i7-9750H (2.60 GHz) CPU with 32 GB of RAM. Although the segmentation time was largely dependent on data quality and user experience, the semi-automated segmentation method with CE-MRA reduced user interaction compared to manual segmentation from scratch. This method currently requires additional time to convert file formats and switch operating platforms. However, this would eventually be reduced or eliminated by software integration. This study focused on LA segmentation, so the roughly outlined LAA was separated from the LA only to obtain a consistent LA segmentation. However, the measured size of the LAA was in agreement with previous studies. Moreover, we were able to clearly observe the shape and border of the LAA with CE-MRA, thanks to the approximately 2.5–3 times higher spatial resolution than with 4D-flow. If we make sufficient efforts to precisely define the LAA, this method will be advantageous for the 4D-flow study of the LAA.

## 5. Limitations

This method required manual input in several steps, so the result may still depend on the operator. The seed-and-grow method required not only seed input but also manual refinement afterwards, and the registration success or failure was determined by visual inspection. An automatic method to judge the registration results based on the flow pattern within the segmentation could be investigated in the future.

All cases were acquired from the database of patients with paroxysmal AF patients prior to ablation. While their 4D-flow was acquired in sinus rhythm, AF induces structural alteration, so they should have had some degree of dilation. Application of this method to healthy controls and persistent AF patients will assess the generalizability of this method.

For the excluded cases prior to volume segmentation, we do not know all the reasons for the low quality, but in most cases it may be an arrhythmia. Although patients were supposed to be in sinus rhythm, it is not uncommon for them to have AF episodes during the 4D-flow scan. Since 4D-flow requires in-line image reconstruction following a long acquisition, it is difficult to detect acquisition and reconstruction errors and rescan the patient during busy clinical routine examinations. Several of the excluded cases presented stripes generated during the reconstruction process. These stripes cannot be corrected during pre-processing. A possible solution is to repeat the in-line reconstruction in the scanner using the acquired raw data. However, this task may be difficult to perform in high-demand clinical routine examinations given the limited time during the scan sessions and the qualifications required to perform this advanced task in the scanner.

In about a quarter of cases, registration failed even after the second attempt. Precise setting of ROI in both CE-MRA and PC-MRA segmentation, for example, contouring along the entire heart, may improve registration. However, it will add another operator manipulation and processing time; therefore, it was not considered. In this study, we only used time-averaged PC-MRA for signal-to-noise ratio, but this method smooths the moving structure to make a difference with the CE-MRA image. Registration with PC-MRA of the individual time phase or the average of a short period may be applied in the future.

Our methodology showed a DSC of 0.77 and a ASSD of 2.7 mm which indicates noticeable differences between the manual LA segmentation from the PC-MRA and the LA segmentation from the CE-MRA. The resampling process into a lower resolution may increase the matching error between volumes. This is an important limitation that needs to be addressed in future work of the study.

Flow was evaluated using mean velocity and stasis. Several other metrics, such as peak velocity, kinetic energy and vortex, have been used in previous studies (30). Based on the assumption that most of the error is in the low-velocity boundary region, we expect that the segmentation difference would not affect peak velocity and vortex at the centre. As kinetic energy is basically a second-order calculation of velocity, the effect of the low-velocity region will be even less. However, investigations on these analyses will prove whether this segmentation method can be used mixed with the traditional method.

Furthermore, this study did not included inter- and intra-variability analysis and scan-rescan assessment. Unveiling the variability and repeatability of the proposed approach must be considered in the continuity of this study.

## 6. Conclusion

The proposed CE-MRA segmentation and registration method can generate segmentation for 4D-flow MRI images. The transformed LA segmentation moderately matches the 4D-flow image and produces analysis results compatible with the conventional segmentation method. The use of this method will facilitate 4D-flow analysis for AF patients by making segmentation easier and overcoming the limitation of resolution and contrast.

## Data Availability

The raw data supporting the conclusions of this article will be made available by the authors, without undue reservation.
